# Pressure gradient measurement to verify hemodynamic results of the chimney endovascular aortic repair (chEVAR) technique

**DOI:** 10.1371/journal.pone.0249549

**Published:** 2021-04-14

**Authors:** Artur Igor Milnerowicz, Aleksandra Milnerowicz, Tomasz Bańkowski, Marcin Protasiewicz

**Affiliations:** 1 Department of Vascular Surgery, 4th Military Clinical Hospital, Wrocław, Poland; 2 Department of Cardiology, Lower Silesia Specialist Hospital of Tadeusz Marciniak Emergency Medicine Center, Wrocław, Poland; 3 Department of Cardiology, Medical University of Wrocław, Wrocław, Poland; Ohio State University Wexner Medical Center Department of Surgery, UNITED STATES

## Abstract

**Purpose:**

The use of the pressure gradient measurements to assess the renal artery flow hemodynamics after chimney endovascular aortic repair (chEVAR).

**Methods:**

The study was a prospective analysis of 37 chEVAR procedures performend in 24 patients with perirenal aortic aneurysm. In all patients the measurement of: distal renal artery pressure (Pd), aortic pressure (Pa), Pd/Pa ratio (Pd/Pa) and mean gradient (MG) between the aorta and the distal renal artery were performed. Measurements were taken with 0.014 inch pressure wire catheter before and after the chEVAR procedure. MG greater than 9 mmHg and Pd/Pa ratio below 0.90 were considered as the measures of a significant decrease in distal pressure that limited flow in renal arteries. The 6 month follow-up computed tomographic angiography (CTA) was performed in all patients to diagnose potential endoleak presence and to verify the patency of the chimney stent-grafts.

**Results:**

All procedures were successful, and no periprocedural complications were observed in any of the patients. The mean gradient values before and after the chimney implantation did not change significantly (6,2±2,0 mmHg and 6,8±2,2 mmHg, respectively). Similarly, no significant change in Pd/Pa values was noted with the value of 0.9 observed both before and after the procedure. All chimney stents were patent on the control CTA. Type Ia endoleak was found in 4 (10.8%) patients.

**Conclusions:**

The application of the described technique seems to be a safe method which allows a direct measurement of renal artery flow hemodynamics before and after chimney implantation during the chEVAR technique. The use of covered balloon expandable stents, ensures the proper blood flow in the renal arteries during the chEVAR technique.

## Introduction

The first report on chimney repair technique for abdominal aortic aneurysm (AAA) was published around 2003 when this method has been described in detail by R. K. Greenberg [1]. Initially, the chimney repair technique was considered an adjunctive salvage procedure used whenever the entry to renal arteries were unintentionally covered during the endovascular aneurysm repair (EVAR). However, shortly after that, the chimney/periscope technique, with the deployment of the stents into abdominal aortic branches, has also been used as a method to retain flow in the renal arteries in patients with juxtarenal or pararenal aneurysms [1–3]. Large studies, involving hundreds of patients, demonstrated that the chimney technique for endovascular aortic repear (chEVAR) was superior to both open surgical technique [4–9] and fenestrated endovascular aneurysm repair (fEVAR) [10–12]. However, chEVAR poses also some challenges for the operator including patient qualification [13], selection of an appropriate device, retaining flow in the stented vessels, and management of potential complications, such as endoleak.

To the best of our knowledge, none of the published studies analyzed the influence of chEVAR on the hemodynamics of flow through the renal arteries. Presence of the stent-graft and its pressure onto the arterial wall brings a potential risk for impaired blood flow and stenosis of visceral vessels [14–16]. Functionally significant stenosis of the stented arteries may lead to dysfunction of the supplied organ. This problem is particularly common in the case of the renal arteries, the stenosis of which may predispose to the development of renal hypertension, exacerbation of preexisting hypertension and impairment of kidney excretory function [17, 18]. The fact that the recanalization of occluded renal chimney stent-grafts can frequently be challenging [19, 20], puts particular emphasis onto an appropriate technique of chimney graft implantation, which would provide adequate patency of the vessel.

The aim of this study was to analyze the influence of chimney EVAR procedures on renal artery flow hemodynamics. The analysis was based on changes of blood pressure in renal arteries before and after aortic stentgraft and chimney stents implantation.

## Materials and methods

### Study population

The study was a prospective analysis of the data collected in 2016–2018. All participants of the study were considered as high-risk patients for open surgical AAA repair (>5% predicted mortality in P-POSSUM scoring system) because of the aneurysm anatomy, comorbidities (ischemic heart disease, congestive heart failure, previous myocardial infarction, coronary stent or bypass, chronic pulmonary disease), history of past open abdominal surgeries, and type Ia endoleak after a previous stent-graft implantation (EVAR). All patients gave their written informed consent for proposed surgical treatment, and the protocol of the study was approved by the Wroclaw Medical University Bioethics Committee (decision number KB-71/2020).

### Computed tomographic angiography

During qualification for the chEVAR, the aorta and AAA were examined using a 0.625mm-slice computed tomographic angiography (CTA). The examination included the thoracic and abdominal aorta, as well as the iliac arteries. The measurements were taken with Osirix Dicom Viewer software for the processing of CTA images. Qualification for the chEVAR and the selection of the stent-graft type were based on current guidelines [21]. The aim of the follow-up CTA carried out 6 months after the procedure was to check for potential endoleak and to verify the patency of the chimney stent-grafts.

### Stent-graft implantation

All procedures were performed at the same center, by the same team of operators. The stent-grafts produced by three companies were used, among them E-tegra^®^ (Jotec, Germany), Zenith Alpha^®^ (Cook Medical, USA) and Treo^®^ (Terumo Aortic, England) (off-the-shelf). The chimney stent-grafts were created with balloon-expandable stents, such as E-Ventus^®^ (Jotec, Germany) and Advanta^®^ (Getinge Group, Sweden). During the selection of the stents, the results of the PERICLES and PROTAGORAS trials were considered; those studies showed lesser incidence of type IA endoleak associated with the gutter between the balloon expandable stent and stent-graft without compromising the long-term patency of the chimneys [22, 23]. The aortic stent-grafts were adjusted in such way that they provided a 20–30% oversizing, seal zone >20 mm, and neck flexure angle no greater than 60 degrees. No self-expandable stents were used.

The chEVAR technique has been described in detail elsewhere [13, 22, 23]. Briefly, the procedure was carried out under local anesthesia of the inguinal and brachial region, in a hybrid operating room with a fixed C-arm. Bilateral femoral access to the iliac arteries was used. Using a rigid guidewire (Amplatz Support Wire^®^, Cook Medical, USA), the main body of the stent-graft was deployed and positioned under angiographic guidance via a diagnostic catheter inserted through the contralateral groin. The chimney stents were deployed using percutaneous brachial access under local anesthesia. The same access was also used to insert the pressure sensor for the pressure gradient measurement (see below). After the baseline pressure gradient measurement, covered balloon-expandable stents were deployed to the renal arteries. During the next stage, the main body of the aortic stent-graft was opened, with its covered part extending at least 15 mm above the upper edge of the renal artery ostium. Then, both renal stents were opened simultaneously in such a way that their orifices were extending approximately 1 cm above the aortic stent-graft. Finally, the contralateral arm of the aortic stent-graft was added. Whenever type Ia endoleak was observed on control CTA, stent-graft angioplasty was carried out with a low-pressure balloon. After the chimney stents were expanded, the pressure sensor was reinserted, and the second pressure gradient measurement was taken.

### Pressure gradient measurement

The measurements were taken with a 0.014-inch PressureWire catheter (St.Jude Medical, USA) in all chimney stented renal arteries. Depending on the aortic anatomy, JR4 or Multipurpose guidewire was used. The guidewire was inserted using percutaneous brachial access and advanced to the ostium of either the right or left renal artery. The PressureWire catheter was introduced into the proximal part of the renal artery and calibrated. After the calibration, the PressureWire catheter was placed in the distal part of the renal artery, and distal pressure (Pd) was monitored. Aortic pressure (Pa) was measured through the guidewire, after its disengagement from the renal artery ostium. Mean pressure gradients between the aorta and the distal renal artery, and the Pd/Pa ratio (MG and Pd/Pa, respectively) were analyzed before and after the stent-graft implantation. The second measurement was taken using the same type of guidewire and PressureWire catheter inserted to the distal segments of the renal arteries. MG greater than 9 mmHg and Pd/Pa ratio below 0.90 were considered as the measures of a significant decrease in distal pressure that limited flow in renal arteries.

After the procedure all patients received dual antiplatelet therapy (aspirin plus clopidogrel, both 75 mg daily) for 3 months followed by lifelong 75mg aspirin according protocol [24].

### Statistics

The statistical analysis was performed using the Statistica 9.0 PL program. To compare the quantitative variables between the two analyzed groups with the normal distribution, the paired-samples t-test was used, and for the others—Wilcoxon signed-rank test. All hypotheses were verified at the statistical significance level p ≤ 0.05.

## Results

### Population

The analysis included 37 chimney implantations to the renal arteries, carried out during 24 procedures in 24 patients. Baseline characteristics of the study patients are shown in Table 1.

**Table 1:** Demographic and clinical characteristics of the study patients.

Parameter	Value
Mean age (years)	71.4 ±7.3
Sex (F/M)	6/18
Diabetes, n (%)	5/24 (20.8)
Hypertension, n (%)	18/24 (75)
Smoking, n (%)	8/24 (33.3)
COPD, n (%)	7/24 (29,1)
Previous MI, n (%)	4/24 (16,6)
Dyslipidemia, n (%)	16/24 (66,6)
Hemoglobin (g/dl)	12.9±1.7

COPD—chronic obstructive pulmonary disease,MI—myocardial infarction.

**Fig 1:**
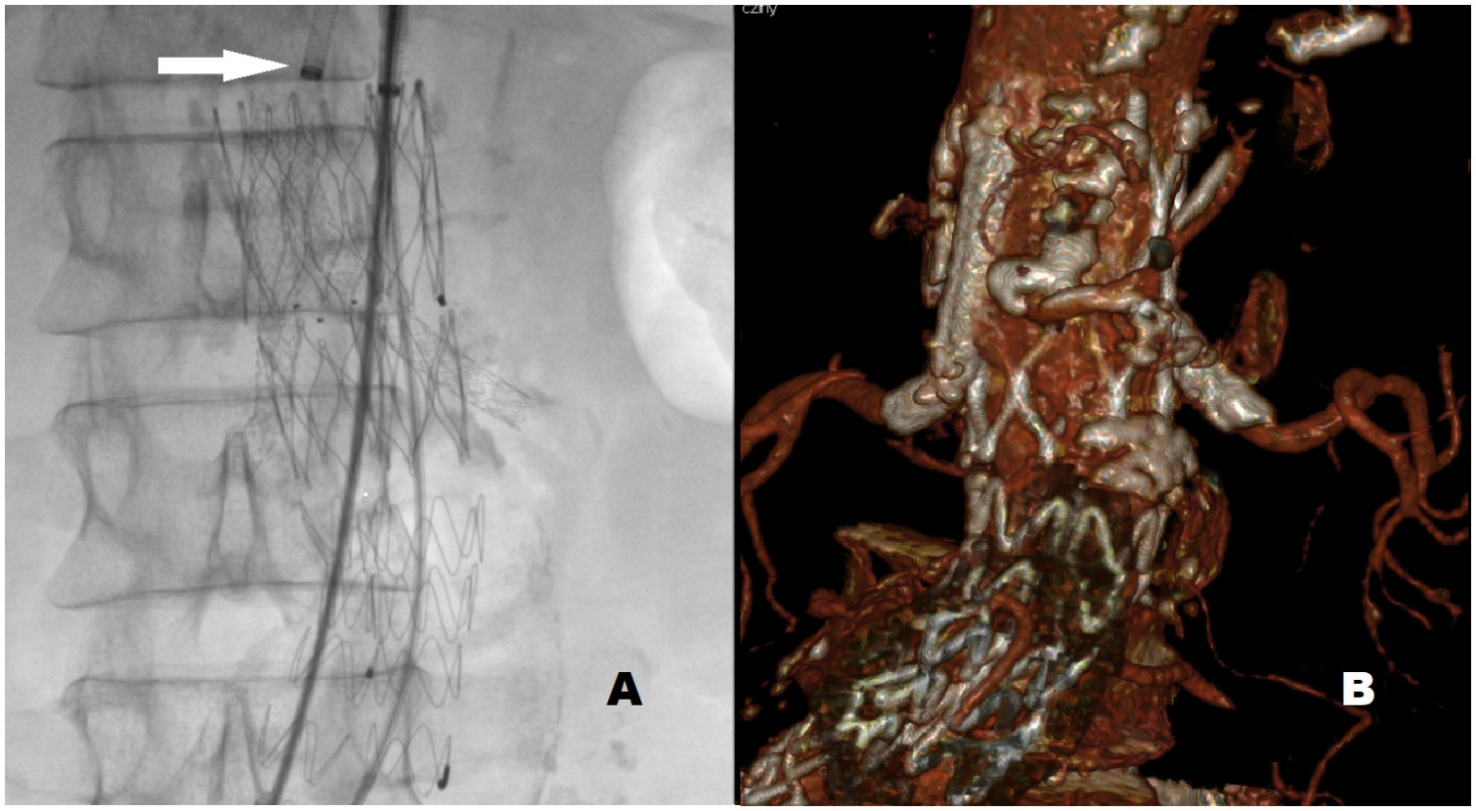
Stentgraft and chimney stents after implantation. A. Angiographic picture during the procedure (arrow -guide catheter for chimney stent implantation and pressure wire delivery). B. Follow up computed tomography after 6 months—patency of both chimney stents.

In the primary chEVAR group, i.e. in patients with contraindications to EVAR associated with comorbidities or aneurysm anatomy, a total of 17 chimneys were implanted to the renal arteries during 11 procedures (in 6 cases to both renal arteries, in 5 cases to single renal artery). Another 20 chimneys were implanted during 13 secondary procedures (in 7 cases to both renal arteries, in 6 cases to single renal artery), carried out because of a type IA endoleak after primary EVAR (secondary chEVAR group). All procedures were successful, and no periprocedural complications were observed in any of the patients. Fig 1A shows an example of angiographic view of aortic stentgraft and renal chimney stents after implantation.

### Computed tomographic angiography

Baseline CTA showed that the mean diameter of the AAAs exceeded 71 mm, and mean length of the aneurysm neck was less than 9 mm; hence, the AAAs did not satisfy the criteria of conventional EVAR. Mean neck flexure angle exceeded 25 degrees, and calcification area at the stent-graft sealing zone was no greater than 25% of the transverse cross-sectional area of the vessel. In none of the cases, renal artery stenosis exceeded 50%.

All chimneys were patent on the control CTA carried out six months from the baseline. The example of follow up CTA is presented in Fig 1B. Type Ia endoleak was found in 4 (10.8%) patients. In three patients, the type IA endoleak co-existed with an increase in the aneurysmal sack diameter. In two cases, the endoleak was controlled successfully through the implantation of branched stent-grafts. One female patient underwent open abdominal surgery with aortic banding at the upper anastomosis, carried out at another center; unfortunately, the patient developed thrombosis in both renal arteries and had to be chronically dialyzed. In another patient, a successful embolization of the gutter between the stent-graft and renal artery was carried out, eliminating type IA endoleak.

Anatomical and clinical characteristics of the study patients are summarized in Table 2.

**Table 2:** Anatomical and clinical characteristics of the study patients.

Parameter	Value
Preoperative mean aneurysm diameter (mm)	71.7±11
Preoperative proximal neck length (mm)	8.6±7
Preoperative suprarenal neck angulation (degrees)	25.1±14
Mean renal artery stenosis (%)	26.2±12.3
Chimney stent diameter (mm)	6.5±0.6
Chimney stent length (mm)	52.1±9.4
Number (%) of patients who received:	
• 1 chimney	11/24 (45.8)
• 2 chimneys	12/24 (50)
• 3 chimneys	1/24 (4.2)
Amount of contrast agent used (ml)	12.9±1.7
Fluoroscopy time (min)	36.7±26.4
Creatinine level before the procedure (mg/dl)	1.2±0.4
Creatinine level 3 days after the procedure (mg/dl)	1.3±0.5
Endoleak Ia incidence after 6 months, n (%)	4/37 (10.8)
Chimney graft occlusion after 6 months, n (%)	0/37 (0)

### Pressure measurement

**Fig 2:**
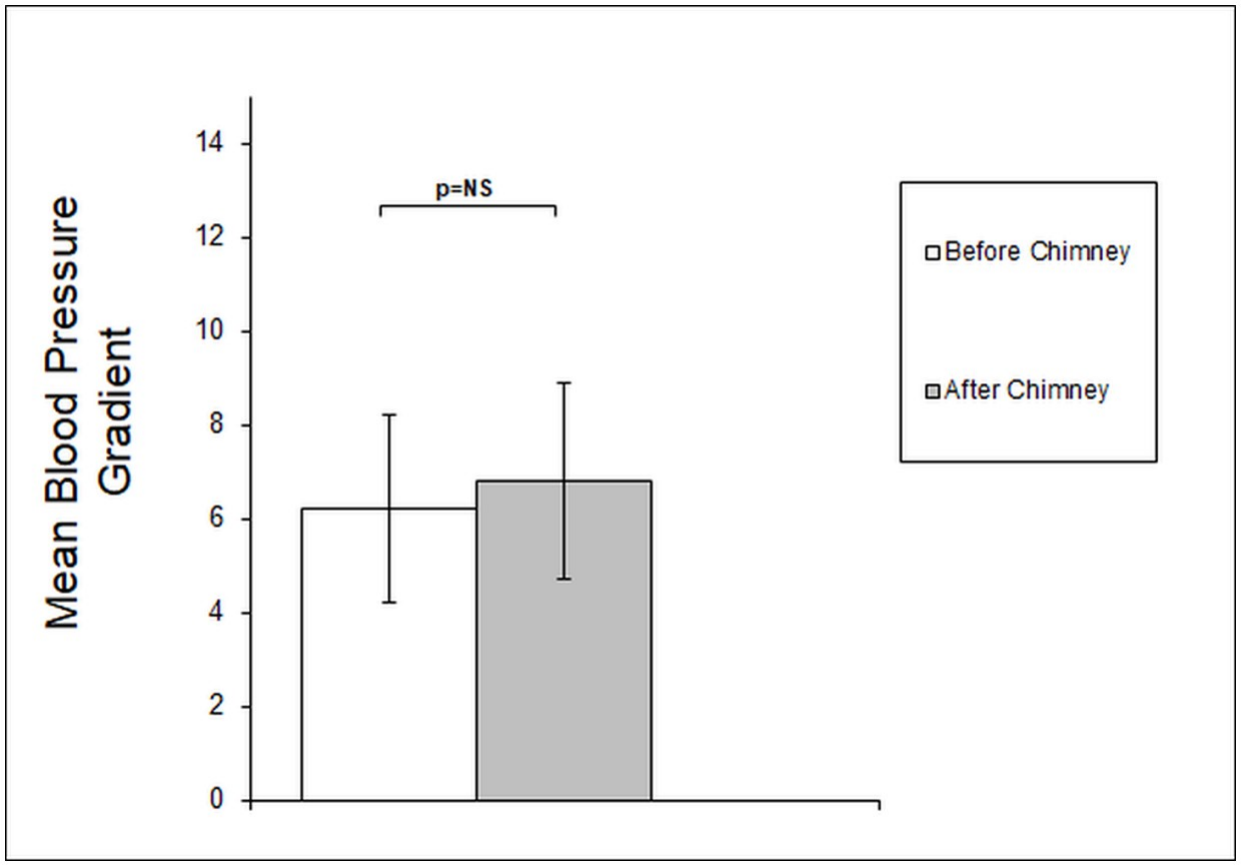
Changes in mean blood pressure gradient before and after chimney implantation. Not significant increase in mean gradient after chEVAR procedure as compared to pre-procedural measurement.

**Fig 3:**
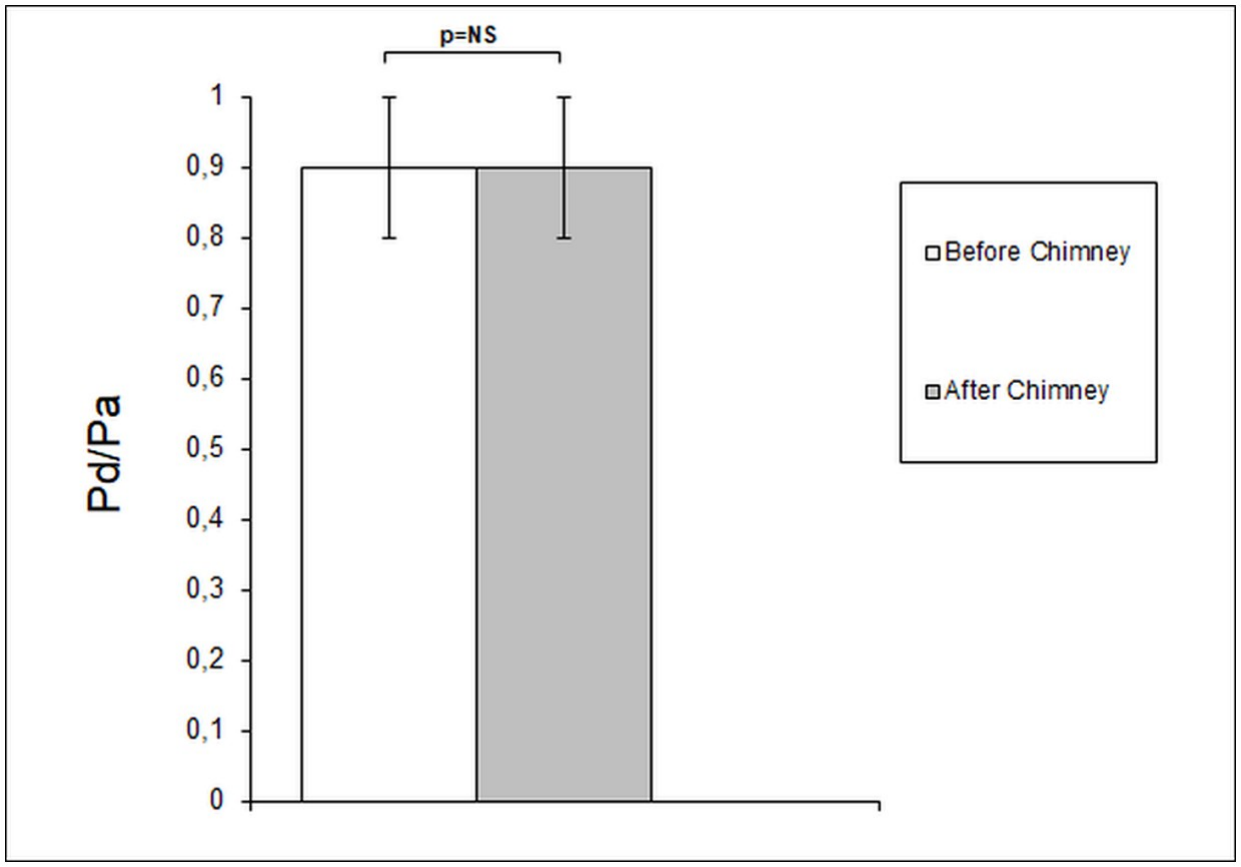
Changes in Pd/Pa before and after chimney implantation. No difference in Pd/Pa ratio before and after chEVAR procedure.

The MG values before and after the chimney implantation were 6,2±2,0 mmHg and 6,8±2,2 mmHg, respectively, and did not differ significantly. Similarly, no significant change in Pd/Pa values was noted with the ratio of 0.9 observed both before and after the implantation procedure. The results of the measurements are shown in Figs 2 and 3.

## Discussion

Our study shows that the renal artery hemodynamic parameters like: distal renal artery pressure, renal artery/aorta pressure ratio or pressure gradient between aorta and renal artery are not affected after renal stent and aortic stentgraft implantation in chEVAR procedure. chEVAR implantation with stable values of MG and Pa/Pa seem not to increase the risk of renal flow disturbances and its potential unfavorable consequences including chimney stent occlusion. The results of CTA follow up in our study, with 100% patency of implanted chimney stents, may support this hypothesis.

In the PROTAGORAS study, the majority of the chimney occlusions occurred within the first two months post-procedure. One of the most important conclusions from this trial was the need for meticulous evaluation of the chimney graft’s morphology and perfusion in completion angiography to recognize and to correct technical problems that might potentially lead to early occlusion [23]. Pressure gradient measurement with dedicated wires has been used commonly in interventional cardiology to assess whether coronary artery stenosis is hemodynamically significant. Clinical usefulness of this method was confirmed in DEFER and FAME trials that analyzed the functional significance of coronary artery stenosis in the single vessel [25] and multivessel coronary artery disease, respectively [26]. Pressure gradient measurement was also used in two studies assessing the significance of renal artery stenosis [27, 28]. Both studies demonstrated that the mean translesional pressure gradient, either at baseline or with hyperemia, provided high sensitivity and specificity in the detection of hemodynamically significant renal artery stenosis. Although administration of dopamine as an inductor of hyperemia in the renal vascular bed increased the sensitivity and specificity of the pressure gradient in the detection of the functionally significant stenosis, the diagnostic accuracy was not substantially better than at the baseline. Given this fact, and considering the complexity of functional testing with dopamine, in our present study, the translesional pressure gradient was determined at baseline. According to literature, the cut-off values that suggest an impairment of renal perfusion range from <0,86 to <0,90 for Pd/Pa [27–29] and from >22 mmHg to >9mm mmHg for baseline MG [27, 28]. We used more restrictive values for both parameters in our present study.

In our opinion, the wire based pressure gradient measurement has many advantages; no additional endovascular access is needed to insert the pressure sensor, the procedure is short and, most importantly, provides information about the flow pressure within the arteries, which makes it an objective examination. What is important the measurement during chimney/stentgraft implantation seems to be very safe, too.

To the best of our knowledge, none of the published studies used an invasive method to analyze the effect of chEVAR on the hemodynamics of flow through the renal arteries. The present study demonstrated that chEVAR was not associated with an increase in the pressure gradient between the aorta and renal arteries, which implies that the chimney stents secured an adequate flow through the renal vessels. This observation constitutes an indirect proof for the effectiveness of chEVAR, confirmed previously in large clinical trials [22, 23]. However, somehow different results were reported by Moulakakis et al. [30]; using CTA image processing software to extract blood flow lumen geometrics, those authors showed that chEVAR and fEVAR contributed to a 43–53% and 15% reduction of flow in the renal arteries, respectively. In their opinion, the relatively long curved length and reduced cross-section of the chimney grafts along their length might contribute to the strong resistance of flow through the renal arteries because of the compression that occurs between the main graft and the wall of the aorta. However, our findings do not seem to support this hypothesis, since a significant increase in the flow resistance would directly affect the pressure gradients in the examined arteries, and no such a phenomenon was observed in our patients. A direct measurement of flow pressure within the examined arteries, makes it a highly objective parameter. Furthermore, it needs to be stressed that the study conducted by Moulakakis et al. included only two patients, which might also influence the results.

We believe that a 6-month period between chEVAR and control CTA was long enough to identify the vast majority of early postprocedural occlusions of the chimney graft. Importantly, this complication was not observed in any of our patients. While it is unlikely that such an satisfactory result was a direct consequence of non-impaired flow through the chimney grafts, in our opinion, it might have been one of the factors contributing to adequate perfusion of the stented vessels during the mid-term observation.

Since the first papers describing chEVAR technique have been published in 2003 [1], this technique underwent multiple modifications and has been a subject of extensive research. High prevalence of juxtarenal AAAs justifies research on their optimal repair method. According to the least optimistic estimates, up to 30% of AAAs may have a hostile neck [11]. Open surgical repair is not always feasible, for example, because of comorbidities or a history of previous open abdominal surgeries. Open AAA repair should be an option solely in patients with <5% risk of periprocedural mortality, and in the era of population aging, the number of patients who satisfy this criterion is gradually decreasing [3–9]. Furthermore, not every center has access to custom-made stent-grafts. The results of the studies that compared chEVAR with fEVAR in terms of type IA endoleak incidence, branch and chimney patency rates and perioperative mortality are highly promising [9–12, 14, 31]. Also, economic arguments seem to favor chEVAR over fEVAR. However, the ultimate conclusion about the superiority of one of these techniques cannot be yet formulated because of the lack of long-term follow-up data.

The indications for chEVAR, as well as the anatomical principles for the selection and fixation of the chimney stent-graft can be clearly defined based on the results of clinical trials, such as PERICLES and PROTAGORAS [13, 22, 23, 32, 33], and we followed them during our present study. Regarding the choice of the device, while both self-expandable and balloon-expandable stents provide similar outcomes in terms of chimney patency rates, the latter were shown to be superior in the prevention of type IA endoleak [22]. However, the choice of a commercially available off-the-shelf stent-graft should be based on individual anatomical conditions in a given patient.

Although our preliminary findings seem promising, we are well aware of the potential limitations of this study, primarily related to small sample size and short duration of the CTA follow-up. We have to admit that study’s sample was not powered sufficiently to detect a significant effect and thus our hypothesis needs to be proved in more statistically powerful studies.

However, results presented in this manuscript might justify further research on the complex hemodynamics after endovascular AAA repair and suggest that pressure gradient measurement might be a promising tool in this setting.
